# Acceptance, use and challenges of digital prevention for arterial hypertension – a qualitative study among patients with high blood pressure in Germany

**DOI:** 10.1186/s12913-025-13284-6

**Published:** 2025-09-01

**Authors:** Eileen Wengemuth, Dunja Bruch, Susann May, Felix Muehlensiepen, Sebastian Spethmann

**Affiliations:** 1 Brandenburg Medical School Theodor Fontane, Centre for Health Services Research, Rüdersdorf Bei Berlin, Germany; 2 Department of Cardiovascular Surgery, Heart Centre Brandenburg, Immanuel Klinikum Bernau, University Hospital Brandenburg Medical School Theodor Fontane, Bernau Bei Berlin, Germany; 3 Faculty of Health Sciences, Brandenburg Medical School Theodor Fontane, Neuruppin, Germany; 4 Department of Cardiology, Angiology and Intensive Care Medicine, Deutsches Herzzentrum Der Charité (DHZC), Berlin, Germany; 5 Charité – Universitätsmedizin Berlin, corporate member of Freie Universität Berlin and Humboldt-Universität Zu Berlin, Berlin, Germany

**Keywords:** Arterial hypertension, Digital prevention, Health apps, Qualitative interviews, Digital divide, Inequality paradox of prevention

## Abstract

**Background:**

Arterial hypertension is one of the leading causes of premature death worldwide. In the care of patients with hypertension, digital tools – such as apps or wearables – can potentially help in prevention to facilitate lifestyle changes or to improve blood pressure control. In hypertension care in Germany, digital applications are only sporadically used by patients and recommended by physicians. This study, therefore, investigates the following questions: What preventive measures do hypertension patients use to manage their condition? What barriers can be identified? How is the use and acceptance of digital technologies for behavioural prevention?

**Methods:**

We conducted semi-structured interviews with 31 patients with arterial hypertension in Germany. The interviews were conducted either in person or by phone and were analysed using Qualitative Content Analysis.

**Results:**

Some interviewees use digital tools for prevention regularly, while others never do. Others have used them in the past or occasionally. Several facilitators and barriers to health behaviours and the use of digital tools were identified. These include the interviewees’ comorbidities, their routines, habits and preferences, as well as their attitude towards technological innovations. Their work situation, their financial resources and the support – or lack thereof – they receive from physicians and from their social environment also play a role. Furthermore, there are differences in the attribution of responsibility among the interviewees. While some attribute responsibility for health to the individual, others emphasise the relevance of aggravating and supportive conditions.

**Conclusions:**

The findings indicate which factors influence the use of digital prevention tools by patients with hypertension. As described in the context of the digital divide and the inequality paradox of prevention, socioeconomic factors play a significant role in both health behaviour and the use of digital prevention tools. Some patients with hypertension may benefit from the use of digital prevention tools if appropriate conditions are created. During implementation, care should be taken to avoid exacerbating existing inequalities.

**Trial registration:**

DRKS00029761 (registration date july 27th 2022).

**Supplementary Information:**

The online version contains supplementary material available at 10.1186/s12913-025-13284-6.

## Background

According to the World Health Organisation, approximately 1.28 billion adults worldwide have arterial hypertension, and it is one of the major causes of premature death. Elevated blood pressure levels over long periods of time can lead to heart attack, heart failure, irregular heartbeat, stroke and kidney failure [1].

In Germany, about one-third of the adult population has been diagnosed with arterial hypertension [2]. Within Germany, there are noticeable regional differences regarding both the prevalence of hypertension and its sequelae, as well as the density of doctors. Especially in the rural eastern regions, the prevalence of hypertension is higher than in the western and/or urban regions. This points to similar inequalities in health and health care that can be observed worldwide, with vulnerable groups being more affected by risk factors and illnesses, in this case, hypertension. This study focuses primarily on the Berlin-Brandenburg region, the capital Berlin, with a raw hypertension prevalence of 23,35% and the surrounding state of Brandenburg, with a raw hypertension prevalence of 35,5% [2]. Additionally, the density of doctors is lower in Brandenburg than in Berlin [3].

In healthcare, as in other sectors, the influence of digital technologies is steadily growing [4]. This includes general eHealth developments, such as online booking of medical appointments, video consultations, and email correspondence with doctors, as well as online courses on diet, exercise, or relaxation. The latter could promote a healthy lifestyle in general, as part of primary prevention, or be specific to certain conditions, in this study’s case, arterial hypertension. However, both are relevant to this study, as the healthy lifestyle suggestions for patients with hypertension are not very different from the general suggestions. In addition, healthy behaviours for hypertensive patients include regular checking and recording of blood pressure, frequent blood pressure monitoring, and adherence to antihypertensive medication [5]. Digital technologies also include mHealth tools such as wearables and smartphone applications. Apps can have different focuses, such as providing health or dietary advice, motivating health behaviour, change, or reminding people of physician or vaccination appointments [6]. For the topic of this study, both general health apps, such as step counters, and hypertension-specific apps, in which patients can record their blood pressure values and/or receive suggestions for health behaviours that benefit their hypertension, are of interest. The general use of health apps is quite common in Germany. 45% of smartphone owners use at least one health application [7]. However, there are also differences in sociodemographic factors, as lower use of health apps is associated with lower income, lower education and older age [8–10]. Other barriers include cost, data privacy, and data security concerns [9]. Facilitators include technology-related aspects such as user-friendliness and perceived usefulness [9, 11], social aspects such as support from family, community members or caregivers [8, 9], and individual characteristics such as digital health literacy [8, 12] and positive attitudes towards technology [8, 13]. Time constraints, the efficiency of digital tools, the simplicity and ease of use, and their integration into clinical workflows were important factors for their involvement in medical consultations [5].

When it comes to apps specifically for cardiovascular health, although the range of available apps is increasing, they are not yet as widely used [14]. Theoretically, digital prevention could potentially compensate for the lower density of healthcare services in rural regions with a high prevalence of hypertension, such as the German federal state of Brandenburg [2]. There is a lack of data on digital tools for hypertension prevention in the German healthcare system [9]. To better understand the state of healthcare provision and to potentially develop possible solutions and personalised approaches, this study addresses the following questions:

- What preventive measures do patients use to manage their hypertension?
- What barriers to preventive measures can be identified?
- How is the use and acceptance of digital technologies for behavioural prevention?

## Methods

### Study design

This study is part of the larger research project “Digital preventive tools for arterial.

Hypertension(DiPaH)”, which identifies structural and individual factors, not only in patients, but also in other stakeholders, that influence the use of digital preventive tools in patients with arterial hypertension [15].

In this study, we employed an exploratory qualitative approach to investigate the healthcare status and stance of hypertensive patients on digital prevention. We first developed an interview guide, which included questions about the patients’ hypertension-related medical history, their healthcare and treatment, their health behaviours, the social and professional support they receive, and their use of digital or other prevention. The complete interview guide is available in the appendix (see Additional File 1). Additional sociodemographic data, such as age, gender, rural/urban residence, level of education, and comorbidities, were collected with a short questionnaire. This can also be found in the appendix (see Additional File 2).

### Data collection

We conducted semi-structured interviews with patients with diagnosed arterial hypertension. We aimed to recruit a diverse group of patients with hypertension, encompassing a range of demographics, including gender, age, health complications, and treatment status. We approached patients in a heart surgery ward for those with severe complications and general practitioners and other doctors in Berlin & Brandenburg for patients with fewer issues. Additionally, we used personal connections to find younger individuals with hypertension and those less engaged with the healthcare system. The study was also advertised on the university's website and social media.

The only inclusion criteria were a diagnosis of arterial hypertension and willingness to participate in the study. We attempted to ensure as much heterogeneity among the interviewees as possible in terms of sociodemographic factors and comorbidities (see interviewee characteristics below). That is why, during the data collection period, we, for example, increasingly approached females, as previously, more males had participated. The interviewees gave informed consent and received an allowance of 30€. The interviews were audio recorded and subsequently transcribed. Thirty interviews were conducted by EW, a psychologist and researcher; SM, a health services researcher, conducted one. Both are experienced in conducting qualitative interviews.

### Data analysis

The interviews were analysed following Qualitative Content Analysis [16, 17] using MAXQDA software. The coding frame was developed based on the first ten interviews and then discussed with the entire project team, after which it was revised. The complete coding frame can be found in the appendix. The codes were partially developed deductively, following the research questions and the questions asked in the interview guide. Partially, we developed the codes inductively depending on what the interviewees brought up as relevant topics in the interviews. Afterwards, the coding frame was applied to the following 21 interviews. The interpretation was performed collaboratively by the research team.

To present the results in this manuscript, we selected representative quotes from different interviewees and translated them. When writing the manuscript, we followed the Consolidated Criteria for Reporting Qualitative Research [18].

### Ethical considerations

The study was approved by the data protection officer and by the ethics committee of Brandenburg Medical School Theodor Fontane, Reference ID: E-02–20220620. All interviewees participated voluntarily, received information about the study, its purposes and methods, and data protection and gave written informed consent for the usage of their data. The recorded interviews were anonymised after transcription.

## Results

### Participants’ characteristics

We interviewed thirty-one patients with diagnosed arterial hypertension between August 2022 and February 2023. The interviews were conducted either in person or by telephone and took between 15 and 35 min. Two interviews with severely ill patients who had just undergone surgery were kept even shorter. The interviews became shorter over the months of data collection, which suggests an increasing data saturation. Thirteen of the interviewees were female, and 18 were male. The age span ranged from 31 to 85 years, with many individuals in their 50 s and 60 s. Fifteen interviewees were from the densely populated capital Berlin, and 12 were from the primarily rural, sparsely populated eastern federal state of Brandenburg. Four of them were from other federal states. Their educational levels were somewhat less heterogeneous than we had intended (see discussion): 19 interviewees held a completed university degree, three had completed 13 years of schooling (grammar school diploma), and eight had undergone nine or ten years of schooling. All but two of them were taking antihypertensive medication. An overview of the participants'characteristics is presented in Table 1.

**Table 1:** Overview of participant characteristics

Interviewee	Age	Gender	Region	Education	Devices	Medication	Cardiovascular risk
W101	67	male	city	university	5 +	yes	medium
W102	59	male	city	university	3	yes	medium
W103	67	male	town	university	4	yes	high
W104	67	male	town	10 years	3	yes	high
W105	63	female	town	10 years	3	yes	high
W106	59	male	rural	university	5 +	yes	high
W107	57	male	town	10 years	3	yes	medium
W108	67	female	city	university	3	yes	medium
W109	68	male	town	university	5 +	no	medium
W110	63	male	town	10 years	4	yes	high
M101	66	female	town	13 years	4	yes	low
W111	30	male	rural	university	5 +	yes	low
W112	35	female	city	university	3	no	low
W113	57	female	city	university	5 +	no	low
W114	69	male	city	10 years	4	yes	high
W115	85	female	city	university	2	yes	high
W116	73	female	city	university	4	yes	low
W117	65	male	rural	university	5 +	yes	high
W118	51	male	city	university	5 +	yes	medium
W119	67	male	city	university	5 +	yes	low
W120	64	male	city	university	4	yes	medium
W121	64	male	city	13 years	4	yes	low
W122	56	female	city	10 years	5 +	yes	low
W123	66	female	city	10 years	3	no	low
W124	59	female	town	9 years	1	yes	low
W125	31	male	city	university	2	yes	medium
W126	57	female	town	13 years	4	yes	low
W127	43	female	city	university	5 +	yes	low
W128	48	female	city	university	4	yes	low
W129	80	male	rural	10 years	4	yes	low
W130	31	male	city	university	3	yes	low

## Overview of categories

We developed the following main categories in the analysis:

- Use of digital preventive tools
- Hypertension-related health behaviour
- Supporting and hindering factors for the use of digital preventive tools and health behaviour
- Attribution of responsibility

### Use of digital preventive tools

Some interviewees regularly use digital preventive tools, especially apps and wearables, to monitor their blood pressure as well as for other health behaviours, mostly exercise. They described these tools as increasing their motivation and helping them to keep track of their blood pressure values and their health behaviours. While digital prevention for arterial hypertension could theoretically include various tools, such as online exercise courses and video consultations, the focus in the interviews was mainly on apps and wearables.

In contrast, other interview partners never use digital prevention tools. Some have heard of them but are not using them themselves, and others are not even aware that they exist.

Yet other interviewees have previously used digital preventive tools, use them occasionally or are interested in trying them, as the following examples illustrate.

Here, an interviewee describes having previously used an app and her reasons for no longer using it.

I: Some people have an app…

*W128: I used to have that too. But to be honest, I found that really unreliable. I had a health app for a while in the beginning. But the problem was that at some point, there was an update and then all the data were lost. And since then I am not doing that any more. (W128, 66)*

In the next interview excerpt, another interviewee describes occasionally using an app.

I: Some people use digital tools, for example they have apps on their phones…

*W108: I do not have that, but I use that whenever I go to the sea with my daughter. She has something like that. I am not that well versed in that. She always has that with her. With these 10.000 steps. (W108, 43–44)*

We have grouped these three forms together because they indicate that interviewees are potentially open to and might benefit from digital preventive tools.

### Hypertension-related health behaviour

Differences also emerge regarding blood pressure-related health behaviours, particularly in terms of reported difficulties or successes in implementing these behaviours. Interviewees primarily discuss diet and exercise, and some also address relaxation techniques. Regularly checking and recording blood pressure can also be considered a relevant health behaviour. However, some interviewees state that measuring their blood pressure too often makes them anxious, and they have agreed with their doctors not to check daily.

Some interviewees report successful health behaviours, for example, eating in accordance with their doctors’ suggestions, whereas other interviewees mention difficulties in implementing health behaviours they consider beneficial for their hypertension. For example, some bring up knowing they drink too much alcohol, or they do not exercise as much as they believe they should. We developed a third subcategory to account for cases in which interviewees report something as beneficial for their blood pressure that does not appear to be consistent with dietary guidelines, as well as instances in which interviewees mentioned both successes and difficulties in putting health behaviours in practice.

Some interviewees link their health behaviour to their use of digital prevention tools; for instance, they perceive using apps or wearables as helpful reminders for exercising. Others, in contrast, do not make this connection and see digital prevention as unrelated to a healthy lifestyle.

### Facilitating and hindering factors for the use of digital prevention and hypertension-related health behaviour

**Fig. 1:**
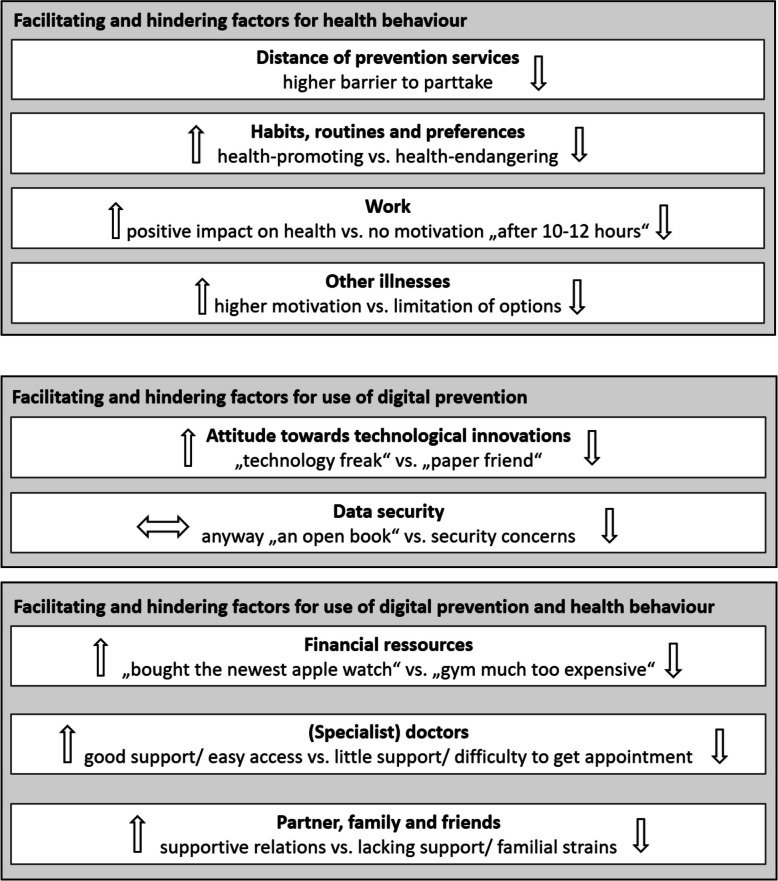
Overview of facilitating and hindering (indicated by arrows) factors for digital prevention use and/or health behaviour

Several facilitating and hindering factors can be identified regarding health behaviours and the use of digital preventive tools. Some of these are specific enablers and barriers for hypertension-related health behaviour, and others for the use of digital preventive tools. Many of them, however, can play a facilitating or hindering role for both. An overview of these factors is presented in Fig. 1.

#### Attitude towards technological innovations

The interviewees’ attitudes towards technological innovations play a role in their use of digital prevention. While some describe themselves as being generally very interested in any new technological developments, always keeping up to date with new devices and functions and having no difficulties using them, others are more sceptical towards or uninterested in technological innovations. For example, one interviewee describes himself as a “technology freak” (W118, 44), whereas another states he is “a paper friend” (W102, 35) and likes to write by hand.

Some also report difficulties using technical devices. Thus, the attitude towards technological innovations can—depending on its form—either facilitate or hinder the use of digital preventive tools in the management of arterial hypertension.

#### Data security

Some interviewees report that they are not using certain apps due to concerns about data security. However, only very few participants mention this topic at all. One participant, on the other hand, reports that she is not bothered by data insecurity because she is “anyway an open book” (M101, 73). In most interviews, however, the topic did not come up. Thus, data security appears to be a relevant factor for some, but by no means for all, people.

#### Financial resources

The interviewees’ financial resources play a role in both their use of digital prevention and their health behaviours related to hypertension. For exercise in particular, their financial resources influence how easily they can participate in a sport that appeals to them. One interviewee reports that for him, being on a minimum wage, “gym (is) much too expensive” (W104, 55). Others, on the other hand, recount that paying for gym memberships or sports courses is not a problem for them. Two of them even have their own pool for swimming. In the context of healthy eating, the issue of financial resources was not raised in any of the interviews; however, it is probably worth exploring. Regarding the use of digital prevention, it became clear that limited storage space on older and cheaper smartphones is relevant for the use of apps and that wearables are always paid for by the users themselves. For example, one interviewee recounts that “following my cardiologist’s recommendation, I have bought the newest Apple Watch” (W117, 17) to monitor his pulse values and to send them regularly to the cardiologist. It is likely that the cardiologist would have been thoughtful enough not to recommend this to a less well-off patient, but consequently, the monitoring would not have been as constant.

#### Access to and support by (specialist) doctors

As seen from the example mentioned above, a supportive physician can positively impact the use of digital preventive tools by suggesting, explaining, and integrating their use into treatment. Several interviewees indicate that they would be willing and interested in using digital prevention if their physician recommended it, and/or that they would appreciate having the blood pressure readings they recorded on their smartphones reviewed by their physician. Regarding health behaviours, it also matters whether physicians inform patients about the beneficial behaviours for their blood pressure and help them find strategies to implement these. Many interviewees mention that their physicians have never informed them about health behaviours that could potentially improve arterial hypertension.

Some interviewees report difficulty getting an appointment with a specialist (cardiologist), especially in rural Brandenburg. One person mentions having had to wait four months and then only getting an appointment two hours’ drive away. Others, especially in urban areas, do not recount having such problems.

#### Partner, family and friends

Partners, family and friends also play a role in both hypertension-related health behaviour and the use of digital preventive tools. Some interviewees describe their significant others as very supportive, reminding them to eat healthy, eating healthy together, encouraging them to exercise, or exercising together. Older interviewees, in particular, mention that their children have installed apps on their smartphones to monitor blood pressure or count steps, bought them wearables, and/or shown them how to use them. Also, the interview clip shown above illustrates this. On the other hand, other interviewees described loneliness and a lack of support as detrimental to their health. Some also reported that familial problems, such as family members with mental illnesses, put additional strain on them and made it difficult to implement health routines.

#### Distance of prevention services

If prevention services, such as sports or nutrition courses, are far away and difficult to access, this prevents some interviewees from consistently participating. Potentially, digital prevention could compensate for this. However, this was not evident in the interviews because interviewees who mentioned accessibility as a problem did not use digital preventive tools more. It should be noted that the speed of internet connections in rural areas in Germany is not always sufficient [19].

#### Habits, routines, preferences

Interviewees who have turned advised health behaviours into a routine, who were already prior to developing hypertension in the habit of behaving that way or who have preferences for and enjoyed these behaviours find it easier to keep these up. For instance, interviewees who have always eaten a lot of vegetables from their own garden or generally enjoy exercise report less difficulty incorporating exercise into their daily routines. On the other hand, interviewees who recount drinking alcohol on a regular basis and/or preferring to eat a lot of meat, find it more difficult to get rid of these habits. Some interviewees report using digital tools to implement routines, such as setting reminders.

#### Work

The interviewees’ work situation also plays an important role in their health behaviour. Most of them see their work as not beneficial to good health, mainly because they work sitting down all the time. Some also report that their work situation prevents them from exercising after work because they work very long hours, often work overtime, and are exhausted by the end of the day. One interviewee mentions that she finds it difficult to establish healthy routines or attend sports courses because she works shifts and her rhythms are constantly changing. Stress is also mentioned as a detrimental factor, especially for cardiovascular health. A few interviewees also see their work situation as beneficial to their health (behaviour), because they work outdoors and/or can exercise at work, receive support and understanding from colleagues or enjoy their work. Several interviewees are also either unemployed or retired.

#### Other illnesses

Many of the interviewees have other conditions in addition to the diagnosis of arterial hypertension. Some of them also have cardiovascular diseases, possibly sequelae of the long-standing hypertension. For some interviewees, their other health conditions may serve as an additional motivation to adopt a health-conscious lifestyle and can be considered an incentive. For others, however, their comorbidities lead to numerous restrictions, such as limitations on the physical activity they can perform.

### Attribution of responsibility

This category has been developed inductively because it emerged as a recurring theme in the interviews. While some interviewees attribute responsibility for health, especially blood pressure, to the individual, others emphasise the relevance of aggravating and supporting conditions.

For instance, some self-critically state it was “their own fault” (W104, 37 W124, 92) that they have arterial hypertension because they have failed to change their lifestyle. Another interviewee states that he believes patients who are overweight, smoke, drink and eat an unhealthy diet should pay higher health insurance premiums. He attributes his healthy lifestyle primarily to his aspirations and ambition (W117, 55, 85). In contrast, other interviewees mention the importance of social support, or lack thereof, and the role of quality connections in access to specialist doctors, emphasising people’s living conditions.

## Discussion

The study aimed to investigate the use and acceptance of (digital) prevention tools from the perspective of patients with hypertension and to identify relevant barriers, especially in the German region of Berlin-Brandenburg.

## Key findings

We found that the use of digital prevention tools, mostly smartphone applications, varies considerably, with some patients using them regularly and others not at all. Some patients were open to using them, used them occasionally, or had previously used them. Patients who regularly used digital prevention tools described them as especially helpful for their hypertension-related health behaviour and for monitoring their blood pressure values. However, it should also be noted that there are patients who do not express a need for digital prevention tools to support their health behaviour. Several factors could be identified that hindered or enabled the use of digital prevention tools and/or health behaviours, such as physician support versus lack of support, partner and family support, sufficient versus low financial resources, and healthy versus unhealthy habits, routines, and preferences.

## Comparison with prior work

Many of the findings on hindering and facilitating factors are in line with previous research from e.g. health psychology research on health behaviour change [20] or social support theory [21].

We found initial evidence that hypertensive patients with fewer financial resources tend to make less use of digital prevention tools. These findings can be understood in the context of the so-called digital divide [22]. For example, research on the digital divide in healthcare suggests that, as mentioned above, the use of health apps is related to income, and that people with lower socioeconomic status tend to have lower e-health literacy [22]. Regarding access, this includes the availability of necessary infrastructure, such as internet connectivity and access to smartphones or wearable devices. Our findings reveal that limited access persists in rural areas, exacerbating inequalities in digital health adoption. Concerning usage, we found that digital literacy and attitudes toward technology significantly influence the effective use of digital tools. Older participants or those with lower education levels often lacked the confidence or skills to engage with digital health solutions, highlighting a usage gap. Lastly, regarding assimilation, the degree to which digital tools are integrated into patients'daily health management routines varied. While some participants found these tools highly motivating and effective, others struggled to incorporate them due to competing demands, lack of support, or mistrust of technology.

Our study suggests that patients with lower socioeconomic status were not only less likely to use digital prevention tools but also reported less support from and more difficult access to doctors, especially specialists, even when taking into account that any necessary medical intervention in Germany is covered by (statutory) health insurance. However, this would need to be verified in a larger sample. This would be in line with the inequality paradox of prevention [23]. It could be argued that digital prevention tools can be seen as an all-population approach because they do not target specific, especially vulnerable groups. Less vulnerable and thus less at-risk groups – as is reported by Frohlich & Potvin for all-population approaches – benefit more from them, thus exacerbating risk and health inequalities. As one of the possible mechanisms behind the inequality paradox, Frohlich & Potvin suggest that the different social positions of healthcare providers and vulnerable groups are a contributing factor [23]. In the case of this study and of digital prevention in general, this difference in social position can be assumed for both doctors and app developers. This again emphasises the importance of participatory development.

Attribution of responsibility, sometimes in the form of individualisation – blaming oneself and/or others –, and sometimes in the form of pointing out flaws in the healthcare system, was a theme that emerged in many interviews. It would be relevant to investigate whether this is specifically relevant to hypertension, a condition often associated with individual health behaviours, and whether this is further reinforced by the focus on digital healthcare, especially apps used individually. It would also be essential to investigate whether patients’ attributions influence how they deal with their hypertension. Psychological theories of attribution [24] suggest that internal attribution leads to more proactive behaviour. On the other hand, there is tentative evidence that (perceived) stigma hinders patients’ adherence to their hypertension treatment [25].

## Limitations

One limitation of this study is that many participants were university graduates, likely indicating a higher socioeconomic status. It is likely that more educated patients can relate more to academic research and are therefore more open to participating in it. It thus seems important to consider and to investigate how healthcare research settings themselves may be exclusive. This overrepresentation may have influenced the study in several ways. Participants with higher levels of education are more likely to possess greater digital literacy and familiarity with digital tools. This could lead to an overestimation of the acceptance and usability of digital health solutions among the general population of patients with hypertension. Individuals with higher education levels may also have greater health awareness and be more proactive in adopting preventive measures, potentially skewing the findings toward more positive health behaviours and attitudes. By not recruiting more vulnerable populations, such as individuals with lower education levels, rural residents with limited access to healthcare, or those with lower socioeconomic status, certain barriers may have been underrepresented: Structural barriers like limited internet connectivity, lack of affordable devices, or inadequate support systems may not have been fully explored. Vulnerable populations might have different attitudes toward digital health technologies, including mistrust or reluctance to engage with new tools due to previous negative experiences or low confidence. In addition, as per the focus of our study, patients were primarily recruited from the German federal states of Berlin and Brandenburg. All findings would thus need to be verified in a larger, preferably more representative sample. As part of the larger research project DiPaH, such a confirmatory study will be conducted with a questionnaire survey.

## Conclusion

The study demonstrates that digital prevention tools, particularly apps, can support hypertension management and are perceived as helpful by some patients, especially for improving health behaviour and monitoring blood pressure. However, their usage remains uneven due to barriers such as financial constraints, limited digital literacy, and restricted access to infrastructure. Vulnerable groups, in particular, often lack the prerequisites to benefit from these tools, potentially exacerbating existing health inequalities.

Some individuals with hypertension may benefit from digital prevention tools if appropriate conditions are created. Key facilitators include recommendations and integration by healthcare providers, as well as the support of family members, particularly for older patients. To promote digital prevention effectively, it is essential to expand digital infrastructure, provide training to enhance digital literacy, and ensure tools are accessible and user-friendly. Care should also be taken during implementation to avoid worsening health and risk inequalities.

Future research should prioritise understanding the unique needs of vulnerable populations, investigate how healthcare providers and developers can counteract inequities, and explore strategies for integrating digital tools into routine care. Larger, more representative samples are necessary to verify the study's findings and develop equitable, inclusive digital health solutions that address long-term health outcomes.

## Supplementary Information

Additional file 1. Translated Interview Guide

Additional file 2. Short questionnaire

Additional file 3. Coding frame

## Data Availability

The coding frame, the interview guide, the questionnaire as well as an overview of the participants’ characteristics can be found in the appendix. The entire German-language interviews are not made publicly available as the participants only consented to use in this study.

## Abbreviation

DiPaH: Research project “Digital Prevention for arterial Hypertension
