# Supplementary material for: Acceptance, use and challenges of digital prevention for arterial hypertension – a qualitative study among patients with high blood pressure in Germany
Source: BMC Health Serv Res. 2025 Sep 1;25:1161. doi: 10.1186/s12913-025-13284-6 (PMC12400765; doi:10.1186/s12913-025-13284-6)
Supplement: Supplementary file 1 — Additional file 1. Translated Interview Guide [file 12913_2025_13284_MOESM1_ESM.pdf]

Interview guide - translated
Interviews with persons with arterial hypertension
Module 1 qualitative part of DiPaH (digital preventive measures for arterial hypertension)

**Guiding question**	**Check**	**Follow-up questions**	**Narration questions**
You have high blood pressure, right? How did you notice this and what happened since then?	Warm-up	What kinds of doctors are you seeing?<br>Have you changed your doctors?<br>How did it come to your current medication? How long did take until a good medication was found?<br><br>How do you measure your blood pressure?<br><br>Do you ever have hypertensive crises? Do you have any secondary complications?	Can you tell me more about it?<br><br>How is/ was that for you?<br><br>Can you give me an example?<br><br>How exactly do you mean?
One can influence one's blood pressure and generally one's health through certain behaviours. What about you? What do you do or try to do?	Health behaviour	What about eating habits?<br>What about exercise?<br>What exactly do you do?<br>How ist that working for you?	
Whom or what do you find supportive for this?<br><br>Which preventive measures do you use?	Supporting and hindering factors<br><br>Leading over to topic of preventive measures	What makes it difficult for you?<br>What role do your family and friends play?<br>What role does your doctor play?<br>What role does your work play?<br><br>What about for instance courses or guides?	

Have you ever used digital preventive measures?<br><br>For Instance Apps or Online-Courses or Video Consultations?	Experiences with digital prevention measures<br><br>Motivation for use<br><br>Supporting and hindering factors<br><br>Attitude towards digitalisation	If YES:<br>What exactly have you used?<br>How did you come to use it?<br>What motivated you to use it?<br>How was that for you? What was your experience? What was difficult, if anything?<br>Do you still use that/ take part in that? If yes, why? If no, why did you stop?<br><br>Who or what supported you?<br><br>If NO:<br>Have you ever considered using something like that/ taking part in something like that?<br>What prevents you from doing that?<br>Could you imagine using digital preventive measures? If yes, what exactly? What kind of support would you need?<br>Who would you like/trust to inform you about digital prevention?<br><br>In general, what is your stance on digitalisation?	
(If there is time): We are almost at the end of our interview. A general question:			
In your opinion, how could the health care situation of people with high blood pressure be improved?	Health care needs and wishes	What do you wish for from your doctor's side?<br><br>What do you wish for from your health insurance's side?	

Thank you very much for your time!
