# Supplementary material for: Acceptance, use and challenges of digital prevention for arterial hypertension – a qualitative study among patients with high blood pressure in Germany
Source: BMC Health Serv Res. 2025 Sep 1;25:1161. doi: 10.1186/s12913-025-13284-6 (PMC12400765; doi:10.1186/s12913-025-13284-6)
Supplement: Supplementary file 2 — Additional file 2. Short questionnaire [file 12913_2025_13284_MOESM2_ESM.pdf]

MEDIZINISCHE HOCHSCHULE BRANDENBURG

Short questionnaire DiPaH

Interviewee (CODE): ________________________________

Date: ____

Age:

____

Gender:

O male O female O diverse

Region of residence

O rural region (less than 5.000 inhabitants)

O small town (5.000 – 20.000 inhabitants)

O medium sized town/city (20.000 – 100.000 inhabitants)

O city (more than 100.000 inhabitants)

Federal state: ______________________________________

Highest level of education:

O No general school leaving certificate

O Secondary school leaving certificate (9 years)

O Intermediate school leaving certificate/ Polytechnic school certificate (10 years)

O Abitur/A-levels/ Advanced school leaving certificate (13 years)

O University or university of applied sciences degree

O Other qualification: _______________________

Availability of technical communication devices:

O telephone	O smart phone	O computer / laptop/ notebook
O smart watch / fitness tracker	O tablet	O fax machine
O others, namely:		

What are your current average blood pressure levels?

systolic (mmHG, „upper value“)	diastolic (mmHG, „lower value“)
O < 120	O < 80
O 120-129	O 80-84
O 130-139	O 85-89
O 140-159	O 90-99
O 160-179	O 100-109
O ≥ 180	O ≥ 110

Do you take blood pressure reducing medication?

O yes

O no

When was your hypertension first diagnosed?

O less than a year ago	O 1 – 5 years ago
O 5 – 10 years ago	O more than 10 years ago

Which other illnesses do you/ did you have, aside from your arterial hypertension?

O heart attack/ stroke

O circulatory problems of coronary vessels or leg arteries

O heart or vascular surgery

O diabetes

O elevated cholesterol levels

O chronic kidney illness

Do you smoke?

O Yes, I smoke.

O I used to smoke regularly.

O I have never smoked regularly.
