# Supplementary material for: Acceptance, use and challenges of digital prevention for arterial hypertension – a qualitative study among patients with high blood pressure in Germany
Source: BMC Health Serv Res. 2025 Sep 1;25:1161. doi: 10.1186/s12913-025-13284-6 (PMC12400765; doi:10.1186/s12913-025-13284-6)
Supplement: Supplementary file 3 — Additional file 3. Coding frame [file 12913_2025_13284_MOESM3_ESM.pdf]

Coding frame DiPaH Module 1 qualitative part

**Health behaviour**	This category is about the efforts that the interviewees make to influence their blood pressure through behaviour, healthy eating, exercise, reduction of alcohol and nicotine consumption, relaxation techniques and stress reduction, as well as measuring and documenting their own blood pressure. To what extent do they feel they are successful in these efforts, and what do they find difficult?
Successful health behaviour	Coded when interviewees report what types of health promoting behaviours they successfully implement. NOTE: This is the personal view of the interviewee. However, this is not coded if their statement or understanding of health promoting behaviour obviously contradicts facts.<br>Anchor example:<br>W108: *I eat a lot of fruit and vegetables and no more than two to three times a month I eat any meat at all. Otherwise I eat a lot of fish, salads, so sweets okay, now and then, but everything in moderation.*
Difficulties in practicing health behaviour	Coded when the interviewees report which types of health-promoting behaviour they have difficulties implementing. In other words, where do they not succeed in carrying out a behaviour that they actually expect to have a positive influence on their blood pressure? Or: where do they not succeed in refraining from a behaviour that they assume will negatively influence their blood pressure? NOTE: This is the personal view of the interviewees. However, this is not coded if their statement or understanding of health-promoting behaviour obviously contradicts facts.<br>Anchor example:<br>W106: *Until a year ago, I unfortunately drank a lot of alcohol. That was not so nice. I knew that too.*
Ambivalent statements on health behaviour	Is 1) coded if the interviewees make contradictory or unclear statements about health behaviour, i.e. if they consider something to be health-promoting which probably does not correspond to the facts or if it is unclear whether they actually implement a certain health behaviour or not.<br>Is 2) coded if the interviewees themselves do not clearly classify their health behaviour as either successful or unsuccessful, e.g. report that they successfully implement some recommendations, but have difficulties with others.<br>Anchor example:<br>W107: *Then, for example, I leave out side dishes, potatoes or something like that completely, I make myself a nice steak, and then I fry some vegetables.*

**Use of digital prevention measures**	This category is about the use of digital prevention measures such as apps and wearables. (Online courses could also fall under this category if they are used by interviewees).
Regular use of digital prevention measures	Is coded if interviewees report that they regularly use digital prevention measures such as apps.<br>Anchor example:<br>W101: *This is a blood pressure monitor. You put the cuff on your upper arm, it pumps up the cuff and shows you the two blood pressure values and the pulse value. And I do that every two days and enter it into an app. The good thing about this app is that when I am with Doctor A, I just give him a code and he can access all the data from the last few weeks and months.*
Occasional or previous use, interested in using digital prevention measures	Coded if interviewees report that they use digital prevention measures<br>- occasionally<br>- have used them in the past<br>- are in principle interested in using them<br>Anchor examples:<br>W108: *I haven't, but I do when I go to the Baltic Sea with my daughter. She has something like that there. I'm not that good at it. And then she always has something like that with her. With these 10,000 steps.*<br><br>W107: *I have been doing that. All the time until now, until the 31st (laughs) And then I stopped doing it, because it's no use if no one checks it.*<br><br>W102: *But of course I could imagine that the app would support me and would then take over and I could also imagine using it.*
No use	Coded if interviewees report that they do not use digital prevention measures.<br>Anchor example:<br>W104: *I have not used them.*
**Facilitating and hindering factors**	What makes it more difficult or easier for the interviewees to make use of prevention services or to implement health-promoting behaviour?<br><br>→ partly distinguishable between 1) digital prevention services and 2) non-digital prevention services and 3) more generally for the implementation of health-promoting behaviour. For many sub codes, however, for all three points.

Supporting and hindering factors\Technological affinity or "paper friend" vs. "technology freak"	Main category on attitudes towards digitalization and technology. What is the general attitude of the interviewees? Do they like communication technologies or not? Do they find them easy or difficult to use?
Rejection of or difficulties with communication technologies	Is coded when interviewees report that they are generally not tech-savvy or online-savvy, that they do not like it, find it unnecessary or have difficulties with it.<br>Anchor examples:<br>W108: *I don't want to. If I do, I want to do it live. And then I'd rather go for a walk. So with this online thing, I'm not that into it...*<br><br>W101: *I'm more of a paper person, I write a lot of things down and stuff. So I tend to be a bit conservative.*
Affinity for and easy handling of communication technologies	Is coded if interviewees report that they are generally tech-savvy and/or that they find it easy to use.<br>*Anchor examples:*<br>W118: *Yes, I'm a bit of a technology freak and have more or less tried out everything on the market.*
Supporting and hindering factors\Data security	Main category on the topic of the degree to which data protection concerns or considerations are decisive for the interviewees when it comes to using digital prevention measures.
Data security concerns play a role	Is coded if the interviewees report that data protection concerns play a role in the use of digital prevention services.<br>Anchor examples:<br>W107: *There is also an app for this. (...) They want to know the location data of the mobile phone, so I don't use this app.*
Data security concerns do not play a role	Is coded if interviewees report that data protection concerns are not important to them when using digital prevention services.<br>Anchor examples:<br>A: *But that means that perhaps the issue of data security also plays a role in why you don't use an app?*<br>M101: *No, that doesn't mean that at all. And do you know why not? Because you're an open book for the digital world anyway. (...) So I don't have a problem with that at all.*
Supporting and hindering factors\access to (specialist) doctors	Main category. How easy or difficult was it for the interviewees to get access to medical specialists? How quickly did they get appointments, etc.?

Difficult access to (specialist) doctors	Is coded if the interviewees report that they had difficulties getting appointments with the relevant (specialist) doctors.<br><u>Anchor example:</u><br>W107: *As far as the shortage of doctors and all that is concerned, (it's) a moderate disaster. That was also the reason why I ended up here in hospital, because I simply couldn't find a doctor, meaning a specialist doctor. My GP said (...) he didn't know what to do and (...) wrote me a referral and I tried for four months to get a specialist. Hopeless.*
Easy access to (specialist) doctors	Is coded if the interviewees report that it was/is easy for them to get appointments with the relevant (specialist) doctors.<br><u>Anchor example:</u><br>W118: *I am extremely lucky that I have had professional dealings with a lot of doctors and fortunately I am usually able to get straight to an address that can help me with the first phone call.*
Supporting and hindering factors\Financial resources	Main category. To what extent do the interviewees' financial resources play a role when it comes to putting health behaviour into practice or using digital prevention measures?
Limited financial resources	Is coded if the interviewees report that their financial means limit them in terms of health behaviour and/or digital prevention.<br><u>Anchor example:</u><br>A: *Rehabilitation sport, that kind of thing is financed by health insurance, isn't it?*<br>W109: *I had to pay for it myself. 32 euros a month. And that lasted as long as I got sick pay. And after that, I only had 850 out of 1,100, and then you can't afford something like that any more.*
Sufficient financial resources	Is coded if the interviewees report that they have sufficient financial resources to enable them to engage in health behaviour and prevention.<br>Often reported implicitly, e.g. when the interviewees report that they have not received support from health insurance/pension insurance for something, that they have bought something themselves (e.g. smartwatch, blood pressure monitor), pay for something themselves (e.g. gym).<br><u>Anchor examples:</u><br>W117: *Following my cardiologist's recommendation, I also bought the latest model of an Apple Watch.*<br>A: *The Apple Watch, I assume that you bought it yourself. For the blood pressure monitor, did you get it reimbursed by your health insurance?*

	W117: *No, I've had private health insurance for 30 years and I don't think that... I'll have to look it up, but I bought it myself.*
Supporting and hindering factors\Habits, routines, preferences	This main category is about habits, routines and preferences that play a role in health behaviour.
Health-promoting habits, routines and preferences	Is coded if the interview partners report habits, routines or preferences that they categorise as health-promoting.<br><u>Anchor examples:</u><br>A: *Who or what helps you to be healthy? (…)*<br>W108: *(…) It's not difficult for me, it's never been difficult for me. I'm also used to it from my parents' house.*
Unhealthy habits, routines and preferences	Wird kodiert wenn die Interview-Partner:innen über Gewohnheiten, Routinen oder Vorlieben sprechen, die sie als nicht gesundheitsförderlich einordnen.<br><u>Ankerbeispiel:</u><br>W121: *I used to eat meat every day. When we went out with colleagues from work in the evening, I always liked to eat these three hundred gram steaks.*
Supporting and hindering factors\distance/ accessibility of prevention measures	This main category deals with the accessibility or distance of prevention services insofar as this has an influence on health behaviour. (Also important insofar as digital prevention measures could theoretically provide a remedy).
Hard-to-reach prevention measures	Is coded if the interviewees report that prevention services are difficult to reach and this has (had) an influence on their use.<br><u>Anchor example:</u><br>W103: *My wife and I were on a nutrition course, we also did Weight Watchers for a year and lost weight. But because it wasn't possible in our town, we always had to drive to X every day. And after a year, when we had lost a lot of weight, we gave it up and then it crept back in a bit over time.*
Good reachability of prevention measures	Is coded if interviewees report that they (more easily) use prevention services or (more easily) practice health behaviour because it is easily reachable.<br><u>Anchor example:</u><br>W119: *I swim a lot in summer. We are in the fortunate position of having our own swimming pond.*

Supporting and hindering factors**Work**	This main category is about the role that the interviewees attribute to their work for their health behaviour and the use of prevention services.<br>This can include whether the job itself is more health-promoting or not (e.g. a lot of sitting or varied movements) as well as the influence that work has on leisure time (leaves enough time and energy or not).
Work promotes health, helpful for health behaviour	Is coded if the interviewees report that they perceive their work as health-promoting or that their work enables them to implement health behaviours.<br><u>Anchor example:</u><br>W108: *I have a lot of exercise.*<br>A: *What kind of exercise do you do?*<br>W108: *Well, I go out every day, I work in childcare. I'm always out and about.*
Work does not promote health, hinders health behaviour	Is coded if the interviewees report that their work is/was not conducive to health or that their work has made it difficult for them to behave in a healthy way.<br><u>Anchor examples:</u><br>W103: *It's not like doing sport. But that wasn't possible in my job because I often had to work overtime. To be honest, I lacked the motivation to go out somewhere in the evening when you've been working for 10 or 12 hours.*<br>W102: *I drive a lot for work and I'm not that dynamic in my work either. (...) It always depended on the job, sometimes more, sometimes less, but I have to say that there could be more movement.*
Supporting and hindering factors**other diseases**	This main category deals with the role that other diseases play in the treatment and prevention of high blood pressure.
Other diseases as conducive to blood pressure-related health behaviour	Is coded if interviewees report that other illnesses tend to have a positive effect on their blood pressure-related health behaviour, e.g. that other illnesses have motivated them to do more for their health.<br><u>Anchor example:</u><br>W106: *Now of course you have to go to hospital fit to get through the operation well, yes? And that was another trigger point. Yes, okay, stop drinking, stop now and prepare for the operation. And I managed to lose 20 kilos.*
Other diseases as a barrier to blood pressure-related health behaviour	Is coded if interviewees report that other illnesses tend to have a negative impact on their blood pressure-related health behaviour, e.g. that they are severely restricted in terms of exercise or similar due to other illnesses.<br><u>Anchor example:</u>

	A: *Are there any factors (…) that make it difficult for you to engage in health-promoting behaviour? (...)*<br>W101: *I haven't really thought about it. I mean, because of my disability (...) I'm quite limited. (...) I can't stand for long periods and that is of course a limitation. Maybe it does have something to do indirectly with the high blood pressure.*
Supporting and hindering factors\support from physicians	This main category is about the support experienced by doctors in the treatment and prevention of high blood pressure and in relation to health behaviour. This also includes, but is not limited to, digital prevention measures.
Little support from physicians	Is coded if the interviewees report that they feel little support from their doctors when it comes to health behaviour and prevention (whether analogue or digital).<br><u>Anchor example:</u><br>W106: *A general practitioner like that, he sees the table, takes a tablet, half less, half more. But I would have expected a bit more support here, yes? Maybe that they would say, why don't you give it a try or something? I think that because I've had it for so many years, it was accepted that way, yes? So I can't say that the doctors motivated me to live differently or to change anything, but at some point I just accepted the situation.*
Good support from physicians	Is coded if the interviewees report that they feel well supported by their doctors in terms of health behaviour and prevention (whether analogue or digital).<br><u>Anchor example:</u><br>W127: *So if I were to be more involved, my doctor would be there immediately and do everything I want. So I'm very happy with my GP.*
Supporting and hindering factors\ support from partner and family	This main category is about the extent to which the interviewees feel supported by their family and their partners in terms of health behaviour and prevention (whether analogue or digital).
Good support from partner and family	Is coded if the interviewees report that they receive support from their partnership and/or family.<br><u>Anchor example:</u><br>W103: *We have a pedometer on our mobile phone to see how many steps we have walked here. We use that.*<br>A: *Yes, okay, I see. How did you come to use it?*<br>W103: *Through the children. (...) They also use it and have practically put it on our mobile phones.*
Little or no support from partner and family	Is coded if interviewees report that they do not receive any support from their partnership or family. NOTE: Not coded if they report that they do not want or need any support!<br><u>Anchor example:</u>

	W110: *There are no more people around me. I still have one cousin in town A. And that's all. The others are all where we all end up.*
**Attribution of responsibility**	This main category is about who the interviewees attribute responsibility for (their own) health to, to what extent they emphasise internal factors such as willpower or self-activation and thus attribute responsibility to the individual, or emphasise restrictive or enabling conditions and the relevance of (social) support.
Individualising attribution of responsibility, excluding conditions	Is coded when the interviewees emphasise that each individual is solely responsible for his/her own health and that they attribute successful health behaviour less to favourable conditions or support from the environment and more to internal factors such as willpower. Conversely, a lack of health behaviour is attributed to a lack of discipline or similar rather than to unfavourable conditions or a lack of support.<br><u>Anchor examples:</u><br>W108: *It's up to each person themselves, so you can tell someone and tell them and they still don't do it. It's always about the person themselves, how they take it and how they deal with it, whether they take their tablets.*<br><br>A: *Who or what helps you to look after your health or exercise and diet?*<br>W103: *You can't blame anyone else for that, you actually have to appeal to yourself to do it.*
Externalising attribution of responsibility, including conditions	Is coded if the interviewee refers to restrictive or enabling conditions with regard to responsibility for their own health and health behaviour or emphasises the importance of (social) support.<br><u>Anchor examples:</u><br>W104: *I didn't do any real sport because the gym (was) far too expensive. (...) You always have to take out 1 and 2-year memberships. That wasn't possible on minimum wage.*<br><br>I: *Was your partner also a support for you?*<br>W106: *Yes, absolutely. She always said: "Why don't you eat more salad? Or why don't you eat a bit healthier, not just meat all the time and, above all, why don't you skip a beer?"*
